# Nitrate: A Crucial Signal during Lateral Roots Development

**DOI:** 10.3389/fpls.2017.00485

**Published:** 2017-04-04

**Authors:** Cui-Hui Sun, Jian-Qiang Yu, Da-Gang Hu

**Affiliations:** ^1^National Key Laboratory of Crop Biology, Shandong Agricultural UniversityTai’An, China; ^2^Ministry of Agriculture Key Laboratory of Horticultural Crop Biology and Germplasm Innovation, Shandong Agricultural UniversityTai’An, China; ^3^College of Horticulture Science and Engineering, Shandong Agricultural UniversityTai’An, China

**Keywords:** nitrate, lateral roots, LR development, primary roots, hormones

## Abstract

Root plasticity is an important trait for plants to forage nutrient and adapt to survival in a complicated environment. Lateral roots (LRs) are generally more sensitive than primary roots in response to changing environmental conditions. As the main source of nitrogen for most higher plants, nitrate acting as a signal has received great attention in the regulation of LR development. In general, there are dual effects including stimulatory and inhibitory of low nitrate on LR development; while high nitrate supply has an inhibitory effect on LR development; nitrate heterogeneity also has a stimulatory effect on LR development in ${\mathrm{NO}}_{3}^{–}$- rich zone. Here, we focus on recent progresses in the role of a nitrate signal in the regulation of the LRs development.

## Introduction

Nitrogen (N) is the most essential macronutrient for plant growth. Plants can absorb various forms of nitrogen, including nitrate (${\mathrm{NO}}_{3}^{–}$), ammonium (${\mathrm{NH}}_{4}^{+}$) and organic amino acid/peptides from soil. Nitrate is the primary source of nitrogen for most higher plants especially in higher pH and more aerobic soils (Crawford and Forde, 2002; Masclaux-Daubresse et al., 2010). In addition to its role as nutrient, nitrate has been proved to act as a signal regulating many physiological processes. For instance, it could regulate root systems architecture (RSA), promote floral induction, and relieve seed dormancy in plants (Zhang et al., 1999; Palenchar et al., 2004; Wang et al., 2004; Remans et al., 2006; Marín et al., 2011).

Roots are crucial for perception and uptake of nitrate in plants (Mounier et al., 2014; Léran et al., 2015; Kiba and Krapp, 2016). Lateral root (LR) development contributes considerably to RSA. In general, LRs are more sensitive to variations in nutrient conditions than primary roots (PRs) (Gruber et al., 2013; Tian et al., 2014). Many studies demonstrated that the formation of LR is a post-embryonic event, beginning with the priming of founder cells in the root pericycle. Then a small part of these cells undergoes a series of asymmetrical, anticlinal and periclinal divisions, forming a dome-shaped LR primordium (LRP). After being activated, emerging and elongating, the LRP finally grows into a mature LR (Malamy and Benfey, 1997; Péret et al., 2009; De Smet, 2012; Petricka et al., 2012). In this review, for better understanding, the developmental process of LR is summarized into three stages, including initiation, emergence (formation) and elongation.

**FIGURE 1:**
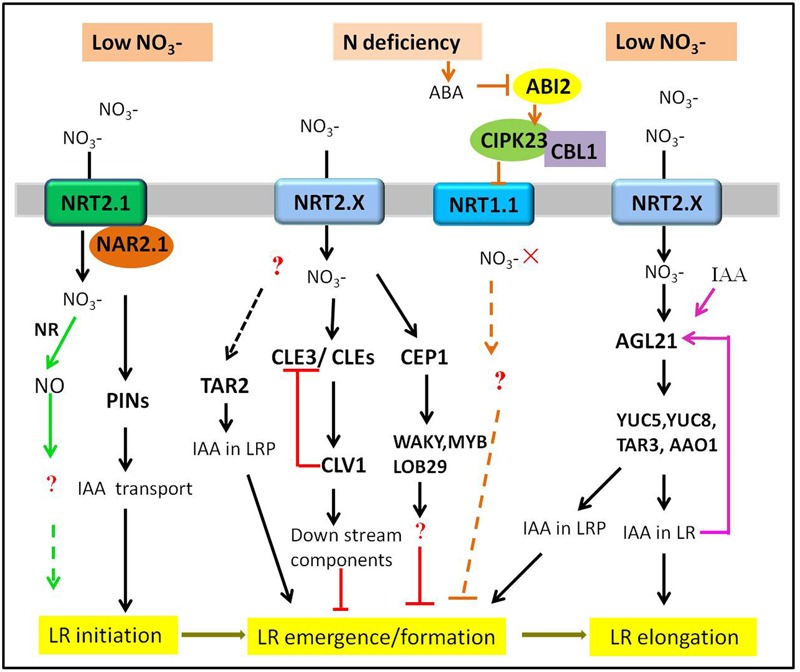
**Multiple signaling pathways regulating the LR response to the low ${\mathrm{NO}}_{3}^{–}$ or N deficiency in plants.** Only those pathways discussed in the present review are depicted. The tip of lines with arrows and horizontal lines indicate the positive and negative signaling steps, respectively. NR, nitrate reductase. See text for further explanation.

**FIGURE 2:**
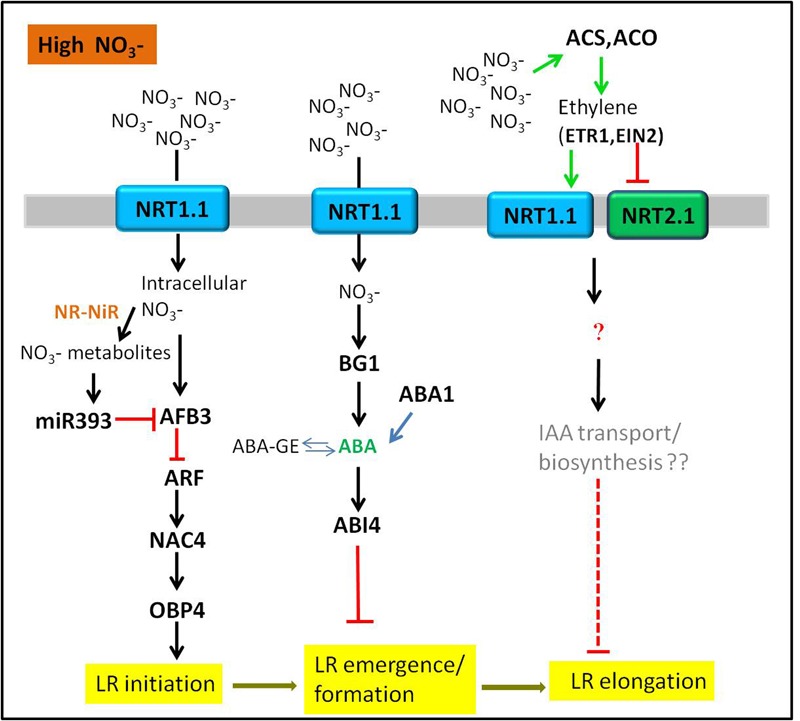
**Sensing of high nitrate signals through hormones-mediated LR development.** The tip of lines with arrows and horizontal lines indicate the positive and negative signaling steps, respectively. NR, nitrate reductase; NiR, nitrite reductase. See text for further information.

**FIGURE 3:**
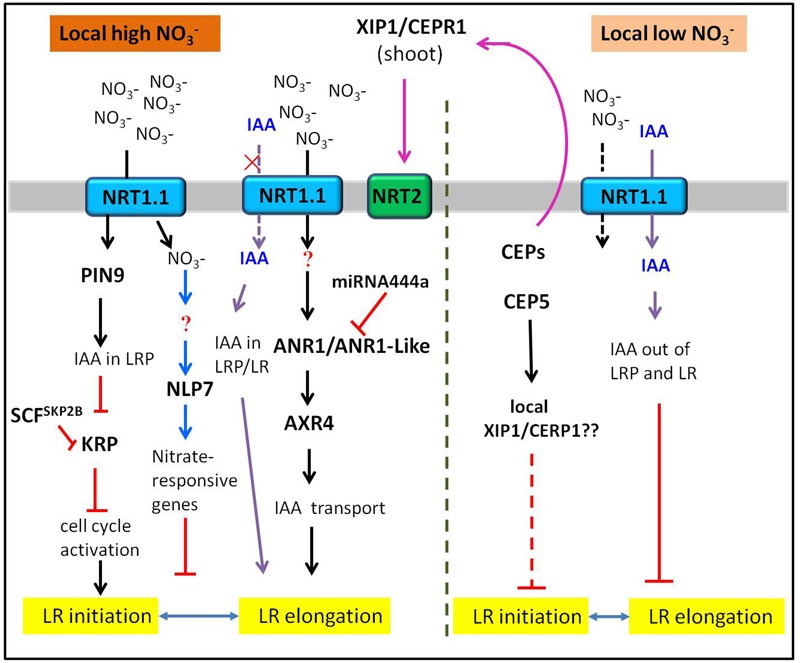
**Schematic drawing illustrating the current knowledge on how heterogeneous nitrate influences the LR development.** The tip of lines with arrows and horizontal lines indicate the positive and negative signaling steps, respectively. See text for further information.

There is increasing studies about the effects of nitrate on LR development in higher plants. However, the nitrate signaling pathways regulating LR development are extremely intricate. Different nitrate signaling pathways have different influences on LR development, depending on nitrate concentration and distribution. Here, we review recent advances in understanding the nitrate signaling pathways that regulate LR development in response to the changes of nitrate supply. The signaling pathways discussed here are illustrated schematically in **Figures 1–3**.

### Dual Roles of Low ${\mathrm{NO}}_{3}^{–}$ on LR Development—Stimulatory and Inhibitory Effects

Previous studies have demonstrated that plants intend to develop a more exploratory root system with longer LRs under N deficiency (López-Bucio et al., 2003; Araya et al., 2014a). However, the effect of N deprivation or low N on root branching varies depending on the nitrogen nutrition situation of the plant itself and the degree to which the plants are stressed (Forde, 2014). Under mild N deficiency, the average length of LR was significantly stimulated. However, when subjected to severe N deficiency, the total length of LR was decreased and LR formation was almost completely absent (Krouk et al., 2010; Gruber et al., 2013). Therefore, it can be predicted that there might be different signaling pathways in the regulation of LR development under N deficiency conditions in plants.

#### The Stimulatory Effects of Low Nitrate on LR Development

Auxin has been proposed as a long-range signal from shoot to root mediating root development in response to nitrate (Forde and Lorenzo, 2001) and there are strong connections between auxin and nitrate signaling, which could cooperatively regulate LR development (Zhang et al., 1999; Gutiérrez et al., 2007; Tian et al., 2008; Krouk et al., 2010; Mounier et al., 2014). A positive effect of mild N deprivation on LR formation in *Arabidopsis* required an auxin biosynthesis gene *TAR2* (tryptophan aminotransferase related 2). *TAR2* can convert L-Trp to indole-3-pyruvic acid (IPyA), which is the first step in the IPyA pathway branching from a Trp-dependent auxin pathway (Tao et al., 2008; Won et al., 2011; Zhao, 2012). Under low ${\mathrm{NO}}_{3}^{–}$ supply, the expression of *TAR2* was up-regulated, resulting in an increase in IAA levels in the developing LRs. However, the *tar2-c* null mutants had much shorter total LR length and fewer visible LR numbers, indicating that mutation of *TAR2* impaired the LR formation (Ma et al., 2014). Therefore, low ${\mathrm{NO}}_{3}^{–}$-stimulated LR emergence depended on root-synthesized auxin in a *TAR2*-dependent manner. However, the components acting upstream of *TAR2* remain unclear.

Another example of the stimulatory effect of low nitrate on LR development was mediated by an *Arabidopsis* AGL17-clade MADs-box gene *AGL21*. The expression of *AGL21* was induced by N deprivation and auxin. The study found that *AGL21*-overexpressing plants produced more visible and longer LRs, while *agl21* mutants showed a reduction on LR elongation under N-restricted conditions. Moreover, auxin biosynthesis genes *YUC5, YUC8*, and *TAR3* were significantly up-regulated in *AGL21*-overexpressing plants and down-regulated in *agl21* mutants (Yu L. et al., 2014). These findings suggested that AGL21 positively regulated LR development by enhancing local auxin biosynthesis in LR primordia and LRs.

In addition, a previous study on rice revealed that knockdown of *OsNAR2.1*, the complementary partener of NRT2.1, inhibited LR formation under low ${\mathrm{NO}}_{3}^{–}$ concentrations, by reducing the expression levels of *PINs* in roots (Huang et al., 2015). This evidence demonstrated that *NAR2.1* played a positive role in regulating LRs development by affecting auxin polar transport under low ${\mathrm{NO}}_{3}^{–}$ conditions. As the author mentioned, the effects of *NAR2.1* on LR formation were most likely by a combination of ${\mathrm{NO}}_{3}^{–}$ uptake and ${\mathrm{NO}}_{3}^{–}$ signaling. NAR2.1 has no ${\mathrm{NO}}_{3}^{–}$ transport function itself, therefore, the role of NRT2.1 may be indispensable in this signaling pathway, which has not been determined.

Apart from the above-mentioned pathways including transcription factors and hormonal signals, more recently, nitric oxide (NO) has been reported to be a key nitrate-related signal to regulate the root system architecture in plants (Chen et al., 2010; Meng et al., 2012; Trevisan et al., 2014). In rice, NO generated by the nitrate reductase (NR) could improve the N acquisition capacity by increasing LR initiation under partial nitrate nutrition (Sun et al., 2015). However, a study in maize supported that NO played a key role in early nitrate perception and could induce a more elevated primary root elongation after low nitrate resupply (Manoli et al., 2014). Therefore, the role of NO in the nitrate signaling pathway needs further evaluation.

In a word, nitrate signaling pathways have a stimulatory effect on LR development under mild N deficiency. The molecular players involved in the pathways regulate different stages of LR development through affecting not only auxin biosynthesis but its transport. Moreover, we speculate that nitrate signaling components act upstream of auxin on regulating LR development.

#### The Inhibitory Effect of Severe N Deficiency

Under severe N deficiency, both formation and length of LRs were inhibited in plants (Gruber et al., 2013). Further studies indicated that they are involved in CLE [CLV3/ENDOSPERM SURROUNDING REGION (ESR)]-related peptides and the CLAVATA1 (CLV1) lecuine-rich repeat receptor-like kinase regulatory module. CLEs and its receptors CLVs control meristem functions and were required for maintenance of shoot apical meristem, respectively, in plants (Clark et al., 1997; Brand et al., 2000; Kondo et al., 2006; Mitchum et al., 2008; Ogawa et al., 2008; Stahl et al., 2009). In addition, *CLE* genes (*CLE1, 3, 4* and *7*) were up-regulated in root pericycle cells when plants were under limited nitrate conditions (<100 μM). What’s more, the *CLE*-overexpressing plants had significantly inhibited outgrowth of LRP and their emergence, while the *clv1* mutants exhibited an increased LR growth. Moreover, overexpression of *CLE3* inhibited LR growth in wild-type plants, but not in *clv1* mutants. The up-regulation of *CLE2, -3, -4*, and *-7* in *clv1* mutants suggested the amplitude of the CLE peptide was feedback-regulated by CLV1 (Araya et al., 2014a,b). In short, CLEs-CLV acted as a regulatory module in the nitrate signaling pathway and negatively regulated lateral root development under N deficiency condition. In addition, C-TERMINALLY ENCODED PEPTIDE (CEP) hormones, 15-amino-acid peptides, are proved to be the negative regulators of root development and growth in plants (Ohyama et al., 2008; Delay et al., 2013; Tabata et al., 2014). A study in *Medicago truncatula* found that the expression of *MtCEP1* was up-regulated by N starvation and overexpressing *MtCEP1* caused the inhibition on LR formation, indicating that MtCEP1 peptide could negatively modulate LR formation under low ${\mathrm{NO}}_{3}^{–}$ concentrations. The further RNA-seq analysis revealed that transcription factors WRKY, bZIP, MYB and homologs of LOB29, SUPERROOT2, etc., may act downstream of MtCEP1 (Imin et al., 2013).

Another mechanism for systemic inhibition of LR development in response to N deficiency was identified. Low nitrate triggered a significant increase in ABA accumulation, and ABA accumulation would inactivate its coreceptor ABI2 (ABA-insensitive 2), a protein phosphatase 2C (PP2C) (Joshi-Saha et al., 2011). ABI2 then interacted with and dephosphorylated the Ca^2+^-sensor subunit CBL1 and the kinase CIPK23 (CBL1-CIPK23) complex, whose substrate was NRT1.1. Moreover, CIPK23 could interact with NRT1.1 in the plasma membrane and phosphorylate NRT1.1, which inhibited the activity of NRT1.1 at low nitrate concentrations (Ho et al., 2009). A recent study reconfirmed the above-mentioned speculative pathway. Mutation of ABI2 leads to the activation of CBL1-CIPK23 complex, then results in reduced root ${\mathrm{NO}}_{3}^{–}$ uptake by repressing the transport activity of NRT1.1 under nitrate deficiency (Léran et al., 2015). As ABA could inhibit LR formation by increasing endogenous ABA biosynthesis in plants (Guo et al., 2009) and the downstream components involved in this pathway are still unclear. Therefore, it is ambiguous whether the negative influence on LR development involving ABA under N deficiency is due to the impaired ${\mathrm{NO}}_{3}^{–}$ signaling effects or ABA physiological function.

In addition, there are some evidences that nitrate transporters with a sensing role are involved in nitrate signaling pathway. NRT1.1 acts as a ${\mathrm{NO}}_{3}^{–}$ transceptor, with a dual transporter/sensor function in signaling pathway (Remans et al., 2006; Ho et al., 2009; Gojon et al., 2011). However, NRT1.1 could control the growth of LRP under the absence of ${\mathrm{NO}}_{3}^{–}$ or low ${\mathrm{NO}}_{3}^{–}$ condition (Bouguyon et al., 2016). While under N-limited condition, *AtNRT2.1* might act as ${\mathrm{NO}}_{3}^{–}$ sensor or signaling component repressing LR initiation, which was independent of its ${\mathrm{NO}}_{3}^{–}$ uptake activity (Little et al., 2005; Remans et al., 2006). Finally, although the definite mechanism is still uncertain, the negative effect of NRT1.1/NRT2.1 on LR development may represent a distinct systemic pathway under low nitrate conditions.

In all, although acting antagonistically and having opposite effects, these systemic pathways coexist to regulate LR development in response to low N. Which pathway taking action depends on the degree of N deficiency that the plants suffered from or their specific environment conditions.

### The Inhibitory Effect of High Nitrate Supply on LR Development

Compared with low nitrate supply, uniform supply of nitrate higher than 10 mM could systematically inhibit both LR branching and LR average length mainly through hormones signaling pathways (Zhang et al., 1999; Tian et al., 2009). There are several possible explanations for the inhibitory effect of high nitrate on LR development.

#### Auxin and Auxin Signaling

Auxin biosynthesis, polar transport, and signal transduction are key players in regulation of LR development (Casimiro et al., 2001; De Smet et al., 2006; Laskowski et al., 2008; Rubio et al., 2009; Krouk et al., 2011; De Smet, 2012). Therefore, the involvement of auxin in the high nitrate concentration-induced inhibitory effect on LR development is of no surprise. Auxin receptor *AFB3* was strongly induced by high nitrate concentration, but was significantly repressed by ${\mathrm{NO}}_{3}^{–}$ metabolites via the feedback regulation by miR393 (Vidal et al., 2010). miR393/AFB3 was identified as a unique module to regulate LR initiation in *Arabidopsis*. The pathway was extended by the founding that nitrate-induction of transcription factors *NAC4* and *OBP4* required the existence of *AFB3*; Similar to the *afb3* mutant, a *nac4* mutant was less sensitive to nitrate-stimulated LR initiation. Meanwhile, the expression of *OBP4* was significantly reduced in *nac4* mutants (Vidal et al., 2013). Therefore, it can be speculated that *AFB3* acts upstream of *NAC4* and *OBP4* to regulate the density of LR. A recent study has extended this pathway in the other direction, nitrate-regulation of *AFB3* and *NAC4* is dependent on the ${\mathrm{NO}}_{3}^{–}$ transport function of NRT1.1, but not its sensing function (Vidal et al., 2014). Except for an auxin signaling pathway, high nitrate regulates LR development also through affecting auxin levels in roots. For instance, when external nitrate concentration was greater than 10 mM, the elongation of LRs in maize was inhibited, which was due to a reduction of auxin translocation from shoot to root in phloem. Therefore, a reduced auxin level in roots resulted in the inhibition of LR growth (Tian et al., 2008).

#### ABA and ABA Signaling

At high concentrations, nitrate inhibited LR outgrowth through ABA signaling (Signora et al., 2001; Vidal et al., 2010). ABA insensitive mutants *abi4-1, abi4-2* and *abi5-1* were less sensitive to the inhibitory effect of high nitrate on LR; and ABA synthesis mutant *abas* produced a significant increase in LR growth, suggesting that the inhibitory effect caused by high nitrate on LR growth was required for both ABA accumulation and signaling (Signora et al., 2001). In addition, genetic analysis revealed that ABA signaling acted downstream of ${\mathrm{NO}}_{3}^{–}$ perception in the regulation of root architecture (Signora et al., 2001). However, a new study demonstrates that high ${\mathrm{NO}}_{3}^{–}$ supply (30 mM) stimulates ABA accumulation in growing roots tips by releasing it from inactive stores via ER-localized β-GLUCOSIDASE1 (BG1), thereby regulating root growth (Ondzighi-Assoume et al., 2016). These data provide a mechanism for ${\mathrm{NO}}_{3}^{–}$-regulated root growth via the regulation of ABA accumulation in the root tip. In conclusion, we can speculate that there is a close interaction between ABA and nitrate signaling, which could jointly regulate LR formation.

#### Ethylene Production and Signaling

Many evidences proved that ethylene inhibited LR development in plants by altering auxin transport and there was crosstalk between ethylene and auxin in regulating LR formation (Ivanchenko et al., 2008; Negi et al., 2008; Lewis et al., 2011). However, the crosstalk between ethylene and nitrate signaling regulating LR development is rarely reported. A previous study found that ethylene was involved in nitrate-dependent LR growth and branching in *Arabidopsis*. High nitrate induced a rapid burst of ethylene in immature LRs. The ethylene burst then upregulated and downregulated the expression of *AtNRT1.1* and *AtNRT2.1* in roots, respectively, resulting in the inhibition of immature LR growth in *Arabidopsis*. In addition, the *etr1-3* and *ein2-1* mutants were insensitive to high nitrate concentrations, suggesting that ethylene signaling genes *ETR1* and *EIN2* might involve in this regulatory pathway (Tian et al., 2009). As there is no related study exploring the players downstream of NRT1.1 and NRT2.1, the mechanism of LR inhibition by ethylene under high nitrate condition becomes obscure. However, IAA and ACC (the precursor of ethylene) could inhibit root elongation synergistically (Stepanova et al., 2007; Swarup et al., 2007). The auxin transport or biosynthesis mediated by ethylene may be the possible explanation under this situation.

### The Stimulatory Effect of ${\mathrm{NO}}_{3}^{–}$ Heterogeneity on LR Development

In response to ${\mathrm{NO}}_{3}^{–}$ heterogeneity (uneven ${\mathrm{NO}}_{3}^{–}$ distribution), most plants could direct preferential LR growth into a ${\mathrm{NO}}_{3}^{–}$ rich zone (Zhang et al., 1999; Giehl et al., 2012). In *Arabidopsis*, local high supply of nitrate specifically stimulated LR elongation (Zhang et al., 1999), while in adult maize, both the length and density of LRs from shoot-borne roots were increased in response to local high nitrate (Yu P. et al., 2014; Yu et al., 2015). The same case was also reported much earlier in barley that the localized nitrate supply could cause an increase in both the numbers and growth rate of LRs (Drew and Saker, 1975). Moreover, the density of LRs on seminal root of rice was increased in response to local ${\mathrm{NO}}_{3}^{–}$ supply (Huang et al., 2015). Many researches have proved that ${\mathrm{NO}}_{3}^{–}$ acted as a signal rather than nutrient to regulate LR proliferation in a localized ${\mathrm{NO}}_{3}^{–}$ rich patch (Zhang et al., 1999; Remans et al., 2006; Krouk et al., 2010). Meanwhile, much progresses have been made to explore the molecular mechanism of stimulatory effect on LR development caused by ${\mathrm{NO}}_{3}^{–}$ heterogeneity.

The first molecular player identified in the signaling pathway is *ANR1*, which belongs to the MADS-box transcription factor family. ANR1 was proved as a positive regulator in the signaling pathway of local nitrate-stimulated LR proliferation (Zhang and Forde, 1998; Gan et al., 2012). However, the realization of full function of *ANR1* requires the presence of nitrate, indicating that there may be other components in this signaling pathway. Later study found that NRT1.1 functioned upstream of *ANR1* in regulating LR elongation, apparently by its role as a ${\mathrm{NO}}_{3}^{–}$ sensor (Remans et al., 2006). The auxin-resistant mutant *axr4* shows no increase in LR elongation after exposure to localized nitrate supply (Zhang et al., 1999), suggesting that *AXR4* gene might be essential for the stimulatory effect of local ${\mathrm{NO}}_{3}^{–}$ signaling. To further explore the members involved in this signaling pathway, we found a recent study on a monocots-specific miRNA, which could target *ANR1* homologous genes and affect LR elongation. *MiR444a*-overexpressing rice lowered the expression of *ANR1-like* genes (*OsMADS-23, OsMADS-27a* and *OsMADS-57*) and impaired the stimulatory effects of localized ${\mathrm{NO}}_{3}^{–}$ on LR growth (Yan et al., 2014). These results indicate that miR444a may participate in the local ${\mathrm{NO}}_{3}^{–}$-signaling pathway by mediating the expression of *ANR1*-homologous genes. Except for ${\mathrm{NO}}_{3}^{–}$ transport activity, NRT1.1 also basipetally transports auxin, which is ${\mathrm{NO}}_{3}^{–}$-dependent (Krouk et al., 2010). Local low nitrate supply facilitates NRT1.1-mediated auxin flux, thus depleting auxin levels in LRP and inhibiting its outgrowth. Conversely, local high nitrate could promote LR development as a result of auxin accumulation in LR primordia and tips by suppressing the NRT1.1-auxin transport activity (Mounier et al., 2014). The role of NRT1.1 repressing LR development on the local low nitrate part in the split root system was similar to its previously identified negative effect under N deficiency. The mechanism discussed here involving in auxin basipetal transport by NRT1.1 may also explain the negative effect by NRT1.1 in the uniform low ${\mathrm{NO}}_{3}^{–}$ condition.

Apart from the NRT1.1-ANR1 involved nitrate signaling pathway, various kinds of new molecular players have been identified guiding different signaling pathway under heterogeneous nitrate conditions. Recent studies on root development in adult maize have been attractive. In adult maize, local high nitrate induces LR initiation in shoot-borne roots of maize by PIN9-mediated auxin efflux and by auxin/SCF^SKP2B^-mediated repression of Kip-related proteins (KRPs), giving rise to subsequent cell-cycle activation in LRP under heterogeneous nitrate conditions (Yu P. et al., 2014; Yu et al., 2015). In addition, the cell type specific RNA-seq experiments revealed that the pericycle cells of brace roots displayed unique transcriptomic reprogramming upon heterogeneous nitrate supply, indicating a specific LR branching pattern on brace roots (Yu et al., 2016).

Besides, CEPs and its membrane-bound receptors are able to control LR growth and development as well in plants (Imin et al., 2013; Roberts et al., 2013; Mohd-Radzman et al., 2016). The low N-produced CEPs act as a long-distance signal through shoot-located XYLEM INTERMIXED WITH PHLOEM 1 (XIP1)/CEP RECEPTOR 1(CEPR1) to up-regulate high-affinity nitrate transporter genes in roots under local high N (Tabata et al., 2014). Further study reveals that auxin-repressive CEP5 inhibits LR initiation probably depending on XIP1/CEPR1 under local high N (Roberts et al., 2016). However, due to restrictions of the extract technique, adaptable methods and approaches on CEPs as well as a lack of a *XIP1/CEPR1* knockout mutant, it is much more difficult to gain a complete understanding of CEP signal regulating LR development.

An important advance in the nitrate signaling pathway has been verified. For example, *NLPs* are a key regulator of nitrate-dependent physiological processes in higher plants (Konishi and Yanagisawa, 2013). One member, NLP7, is not nitrate-responsive at the transcriptional level, but mediates early nitrate sensing and assimilation (Castaings et al., 2009). Further study unraveled that NLP7 played a key role in LR development in response to nitrate. These studies found that the *nlp7* mutants have an increased LR density on the high ${\mathrm{NO}}_{3}^{–}$ side, and are impaired in nitrate sensing and nitrate assimilation. In addition, *nlp7* mutants show severe inhibition of LR growth on both high- and low-nitrate media on split-root plates, indicating NLP7 may mediate local nitrate responses (Marchive et al., 2013; Guan et al., 2014). However, the existing studies indirectly put forward the potential role of NLP7 in regulating LR development under different nitrate availability. It seems that NLP7 has a negative effect on LR development, which still requires further investigation.

## Conclusion and Perspectives

There have been rapid advances in understanding the molecular mechanism that regulate LR growth and development under different ${\mathrm{NO}}_{3}^{–}$ conditions in different higher plants. The signaling pathways discussed here show us the complexity of nitrate regulation of LR development. Not only the redundancy but sometimes even antagonism between different pathways is confusing in understanding, let alone much more unexplored components in some pathways. Fortunately, systems biology approaches including the emergence of new genome-editing technologies such as CRISP-Cas9, as well as the high-throughout RNA sequencing technologies allow for identification of candidate genes, which will lead to significant advances in the future.

Most of the studies cited in this review are mainly focusing on model plants. By comparing, the cereal crops such as maize and rice have more complex root systems than the model plant *Arabidopsis*. What’s more, except the common pathway controlling LR initiation, there are unique root-type-specific pathways in cereal crops, which primarily regulate LR formation. For example, despite the evolutionary differences between *Arabidopsis* and rice, the function of *ANR1/ANR1-like* genes in nitrate regulation of LR growth is highly conserved. So the knowledge of nitrate signaling pathways gained from *Arabidopsis* would be applicable to cereal crops. However, the above-mentioned gene *PIN9* is monocot-root specific, which is highly expressed in LPRs in maize, controlling auxin transport and redistribution triggered by local nitrate supply. Therefore, it is noteworthy that the molecular mechanisms regulating LR development in response to nitrate in *Arabidopsis* and cereal crops sometimes are in common use, however, sometimes may be species-specific.

Plants absorb various types of nitrogen from the soil, however, only nitrate possesses such unique and vital signaling effects on LR development. What’s more, the signaling effect of nitrate is partly far more important than its nutrimental function. Many evidences suggest that nitrate signaling effects exert indispensable physiological meaning for plant growth and adaptation to changing survival environment. For example, under nitrate-limited conditions (low ${\mathrm{NO}}_{3}^{–}$ supply), plants tend to adapt a ‘foraging strategy’ with LR proliferation for more nutrients. By contrast, in nitrate-repleted conditions (high ${\mathrm{NO}}_{3}^{–}$ supply), plant roots adapt an economic saving model with LR inhibition to reserve energy for other usage. However, uneven nitrate distribution in the soil could trigger more LRs foraging more nutrients in ${\mathrm{NO}}_{3}^{–}$-rich zones in order to compensate for the limited N acquisition in ${\mathrm{NO}}_{3}^{–}$-deficient patches. On thinking of this, we couldn’t help admiring plants for their strong adaptability to the environment.

## Author Contributions

C-HS, D-GH, J-QY wrote the manuscript. D-GH provided assistance for further modification of manuscript. All authors listed, have made substantial, direct and intellectual contribution to the work, and approved it for publication.

## Conflict of Interest Statement

The authors declare that the research was conducted in the absence of any commercial or financial relationships that could be construed as a potential conflict of interest.
